# The relationship between chronic obstructive pulmonary disease and non‐small cell lung cancer in the elderly

**DOI:** 10.1002/cam4.2333

**Published:** 2019-06-11

**Authors:** Peng Wang, Min Zhu, Dong Zhang, Xue‐guang Guo, Shu Zhao, Xue‐lin Zhang, De‐long Wang, Chang‐ting Liu

**Affiliations:** ^1^ Department of Medical Oncology, The Second Medical Centre Chinese People's Liberation Army General Hospital Beijing China; ^2^ Department of Respiratory Diseases, The Second Medical Centre Chinese People's Liberation Army General Hospital Beijing China

**Keywords:** cancer management, cancer risk factors, lung cancer

## Abstract

**Objectives:**

Chronic obstructive pulmonary disease (COPD) and NSCLC often coexist and have poor prognoses, but studies investigating the impact of COPD on NSCLC have reported inconsistent findings. The objective of this study was to compare survival between NSCLC patients with and without COPD.

**Methods:**

Medical records were retrospectively collected from 301 elderly patients pathologically diagnosed with NSCLC from the Chinese PLA General Hospital. Ultimately, a total of 200 patients were enrolled in the analysis. The survival rates between the COPD‐NSCLC and non‐COPD NSCLC were assessed using log‐rank and Cox proportional hazard regression analyses.

**Results:**

A total of 117 COPD‐NSCLC and 93 non‐COPD NSCLC patients were enrolled in the analysis. The median overall survival times were 108.5 months in the non‐COPD group and 45.0 months in the COPD group (HR: 2.05; 95% CI, 1.36‐2.97, *P* = 0.0004). After 118 patients underwent propensity score matching, the median overall survival times were 100.6 months in the non‐COPD group and 51.9 months in the COPD group (HR: 1.59; 95% CI, 1.096‐2.64, *P* = 0.0459). The multivariate analysis showed that presence of COPD (HR 1.619, *P* = 0.030), old age (HR 1.007, *P* < 00001), an advanced disease stage (stage Ⅲ HR 5.513, *P* < 0.0001; stage Ⅳ HR 11.743, *P* < 0.0001), the squamous cell carcinoma histological subtype (HR 3.106, *P* < 0.0001), the presence of a cough (HR 2.463, *P* = 0.001) a higher serum carcinoembryonic antigen level (HR 1.001, *P* = 0.023) and higher NRL (HR 2.615, *P* = 0.007) were independent factors that were significantly associated with poorer survival.

**Conclusion:**

A diagnosis of COPD had significant poorer survival outcomes in NSCLC than that of patients without COPD in this elderly population.

## 1 INTRODUCTION

Lung cancer is the most frequently diagnosed cancer and the leading cause of cancer‐related death worldwide. More than one‐quarter of all cancer deaths are due to lung cancer.^1^, ^2^ Chronic obstructive pulmonary disease (COPD) is another pulmonary disease and the fourth leading cause of death, causing more than 2.5 million deaths per year worldwide.^3^, ^4^, ^5^ Cigarette smoke exposure is the common environmental risk factor for both of the diseases. As shown by Potton et al, cigarette exposure causes the bronchial epithelium to undergo several histological changes, which is thought to lead to lung cancer, including metaplasia, dysplasia, carcinoma‐in situ and adenomatous hyperplasia. Sustained pulmonary inflammation and the lung matrix remodeling underlying COPD may be the important triggers for lung cancer.^6^ Vaguliene et al^7^ showed that chronic inflammation was very distinct in patients with lung cancer based on the significantly higher level of C‐reactive protein (CRP). Young and Hopkins suggested that the relationship between COPD and lung cancer was similar to the relationship observed between obesity and type 2 diabetes, in which one disease was pathogenically related to the other.^8^

The survival outcomes between lung cancer populations with and without COPD have been reported in several large‐scale independent studies, but the results are inconsistent. For example, Lee et al,^9^ Gullon et al^10^ and Mina et al^11^ reported that COPD had no impact on the mortality of NSCLC patients. Conversely, Sekine et al analyzed 442 cases of stage IA lung cancer in which the patients underwent complete surgical resection and found that patients with COPD had poorer survival, possibly for the reason of higher incidence of tumor recurrence.^12^ The report from Zhai et al^13^ showed that stage IA‐IIB NSCLC coexisting COPD was related to worse survival by surgical resection. The conflicting outcomes may be affected by complicated clinical features, including age, gender, disease stage, treatment, comorbidities, and smoking status.

Considering these ambiguous results, we performed a study with the aim of analyzing the influence of COPD on the NSCLC prognosis. We report the 15‐year survival outcomes of patients with NSCLC with and without coexisting COPD in our Geriatric Oncologic Center.

## 2 MATERIALS AND METHODS

### 2.1 Study population

This retrospective study was approved by the Ethics Committee of our institution. Clinical information was obtained from the Chinese People's Liberation Army General Hospital electronic file database. A total of 301 patients with NSCLC diagnosed histologically and/or cytologically from January 1, 2000, to May 1, 2015, in the geriatric ward were recruited for this study. 80 patients were excluded because of the lack of pulmonary function. The other exclusion criteria were (a) loss of follow‐up records (n = 11) and (b) coexistence of an end‐stage malignant disease that affected survival (n = 10). We selected 200 of the 301 patients NSCLC patients with pulmonary function tests and complete medical records to conduct this analysis.

The pulmonary function test of each patient conducted closest to the day of the NSCLC diagnosis was selected. COPD was diagnosed by a pulmonary physician if the following criteria were met: airflow obstruction defined as a post‐bronchodilator forced expiratory volume in 1 second (FEV1) ≤70% of the forced vital capacity (FVC). The GOLD (Global Obstructive Lung Disease) classification score was used to classify the severity of airflow obstruction associated with spirometry as mild (GOLD I; FEV1 ≥80% of predicted), moderate (GOLD II; FEV1 = 50%‐79% of predicted), severe (GOLD III; FEV1 = 30%‐49% of predicted), and very severe (GOLD IV; FEV1 <30% of predicted).

Other baseline demographics and clinical characteristics included age, gender, smoking history, smoking index, Eastern Cooperative Oncology Group performance status (PS), TNM stage, histological type, initial treatment method, symptoms at diagnosis, comorbidities, and the serum carcinoembryonic antigen (CEA), neutrophil to lymphocyte ratio (NLR) and D2 dimer levels. The TNM stage was performed using the UICC TNM‐7. The ECOG PS was grouped using a cut‐off value of 2. The histological types were squamous cell carcinoma, adenocarcinoma, adeno‐squamous carcinoma, NSCLC not otherwise specified, and other, which included carcinoid tumor and large cell carcinoma. All NSCLC initial treatment decisions were made by a multidisciplinary team (MDT) that included an oncologist, thoracic surgeon, radiologist, radiation oncologist, and anesthetist. Possible treatment decisions included surgery, radiation, chemotherapy, target therapy, and best supportive care (BSC).

### 2.2 Statistical analysis

Clinical characteristics were analyzed using Fisher's exact test. Descriptive statistics were expressed as the mean ± standard deviation (SD). Independent sample t‐tests and one‐way analysis of variance (ANOVA) were used to compare the continuous variables between the COPD and non‐COPD patients. The Kaplan‐Meier estimate was used to analyze overall survival (OS), and differences were compared using the log‐rank test. OS was defined as the number of days between the treatment and the date of death or the cut‐off date. The independent risk factors for poor survival were analyzed using a Cox proportional hazards regression model. Only significant variables in the univariate analysis were entered into the multivariate analysis. Hazard ratios (HR) and 95% confidence intervals (CI) were estimated. All statistical tests were two‐sided with a threshold of *P* ≤ 0.05 for statistical significance and were performed using Stat View version 9.4 (SAS Inc, Cary, NC, USA).

We used propensity score matching (PSM) to evaluate the comparability between treatment cohorts. Patients' data were anonymized before PSM using covariates including gender, age, moking index, TNM stage, ECOG PS, histologic type, method of initial treatment, symptoms at diagnosis, comorbid diseases, and serum level of carcinoembryonic antigen and D2 dimmer. Matching was carried out at a ratio of 1:1 and a caliper distance of 0.01 without replacement. Matching was performed using a semi‐automated method with the Matching package (version 4.8.3.4) of R (version 3.0.1).

### 2.3 Compliance with ethical standards

All procedures performed in this study were in accordance with the ethical standards of the institutional and/or national research committee and the 1964 Declaration of Helsinki and its later amendments or comparable ethical standards. The present study was approved by the Institutional Ethics Review Board at the Chinese PLA General Hospital.

## 3 RESULTS

One hundred and seven COPD‐NSCLC patients and 93 non‐COPD NSCLC patients were enrolled in the study. The baseline characteristics of the patients before PSM are listed in Table 1. The patients with COPD were significantly older than the patients without COPD (mean age ± standard deviation, 78.6 ± 9.05 vs 74.3 ± 9.6 years; *P* = 0.001). The ECOG PS scores, TNM stage and pulmonary function were not balanced between the two cohorts, and the clinical condition in the COPD group was more likely to be significantly worse than the clinical condition in the non‐COPD group. As shown in Table 1, the pulmonary function tests revealed that the COPD group had a significantly poorer FEV1/FVC (77.6 ± 5.8 vs 59.75 ± 8.3; *P* < 0.001), percent predicted FEV1 (99.17 ± 19.1 vs 72.12 ± 21.0; *P* < 0.001), and FEV1 (2.3 ± 0.6 vs 1.6 ± 0.6; *P* < 0.001) than the non‐COPD group. Additionally, the COPD group included more heavy smokers and fewer surgery candidates. Adenocarcinoma was the most frequent histological subtype in both groups, with 4 (4.3%) cases in the non‐COPD group (large cell carcinoma = 3 and carcinoid tumor = 1) and 6 cases (5.6%) in the COPD group (large cell carcinoma = 5 and sarcomatoid carcinoma = 1) with other histological subtypes. NLR was significantly higher in COPD group. The gender ratio, presence of previous tumors, and serum CEA and D2 dimer levels were comparable between the groups.

**Table 1:** Demographic and clinical characteristics

Characteristics	Patients no (%)		
	Non‐COPD (n = 93)	COPD (n = 107)	*P*
Mean age ± SD	74.3 ± 9.6	78.6 ± 9.05	0.001
Range	50 ~ 91	54 ~ 101	
Age ≥75 y old^a^			
Yes	47 (50.5)	79 (73.8)	0.001
No	46 (49.5)	28 (26.2)	
Gender^a^			
Male	86 (92.5)	103 (96.3)	0.241
Female	7 (7.5)	4 (3.7)	
Smoking index, Pack‐years^a^			
≥20	36 (38.7)	63 (58.9%)	0.004
<20	57 (61.3%)	44 (41.1%)	
Pulmonary function test			
FEV1/FVC, %	77.6 ± 5.8	59.75 ± 8.3	<0.001
Predicted FEV1, %	99.17 ± 19.1	72.12 ± 21.0	<0.001
FEV1, L	2.3 ± 0.6	1.6 ± 0.6	<0.001
FVC, L	3.0 ± 0.7	2.7 ± 0.8	0.002
FVC, %	92.6 ± 19.0	83.9 ± 20.8	0.003
COPD severity			
Mild		48 (44.9)	
Moderate		39 (36.4)	
(Very) Severe		20 (18.7)	
ECOG PS score^a^			
0 ~ 1	57 (61.3)	45 (42.1)	0.007
≥2	36 (38.7)	62 (57.9)	
TNM stage^a^			
Ⅰ	63 (67.7)	57 (53.3)	0.026
Ⅱ	6 (6.5)	3 (2.8)	
Ⅲ	11 (11.8)	29 (27.1)	
Ⅳ	13 (14.0)	18 (16.8)	
Histologic subtype^a^			
Squamous cell carcinoma	19 (20.4)	31 (29.0)	0.344
Adenocarcinoma	54 (58.1)	49 (45.8)	
Adenosquamous carcinoma	1 (1.1)	4 (3.7)	
NSCLC, NOS	15 (16.1)	17 (15.9)	
Others	4 (4.3)	6 (5.6)	
Initial treatment^a^			
Surgery	45 (48.4)	31 (29.0)	0.031
Radiation	17 (18.3)	32 (29.9)	
Chemotherapy	9 (9.7)	11 (10.3)	
Target therapy	15 (16.1)	16 (15.0)	
BSC	7 (7.5)	17 (15.9)	
Previous tumors^a^			
Yes	24 (25.8)	34 (31.8)	0.353
No	69 (74.2)	73 (68.2)	
Serum level of CEA, ng/mL	21.4 ± 139.8	34.5 ± 300.2	0.7
Serum level of D2‐dimer	1.0 ± 1.7	0.9 ± 1.5	0.751
Neutrophil‐lymphocyte ratio	1.8 ± 1.0	3.5 ± 2.0	<0.001

Abbreviations: BSC, best supportive care; CEA, carcinoembryonic antigen; COPD, chronic obstructive pulmonary disease; ECOG PS, Eastern Cooperative Oncology Group performance status; FEV1, forced expiratory volume in 1 s; FVC, forced vital capacity; NLR, neutrophil‐lymphocyte ratio.

a Variable were used to compute propensity scores.

A review of the symptoms and comorbidities at diagnosis are listed in Table 2. A cough was the most frequent symptom in both groups (10.8% in the non‐COPD group *vs* 15.0% in the COPD group; *P* = 0.378), followed by dyspnea and hemoptysis. Notably, 54.8% and 42.1% of the patients in the non‐COPD and COPD groups, respectively, did not exhibit any symptoms in their regular physical examination at the time of the NSCLC diagnosis. Hypertension and cardiovascular diseases were the most common comorbid diseases in both groups, followed by diabetes and cerebrovascular diseases. Patients who had COPD had a significantly higher frequency of cardiovascular diseases (49.5% vs 65.4%, *P* = 0.023) and cerebrovascular diseases (21.5% vs 35.5%, *P* = 0.029). Notably, only 17 patients in the 2 groups did not have other diseases (10 in the non‐COPD group and 7 in the COPD group).

**Table 2:** Symptoms at diagnosis and comorbidities

	Patients no (%)		
	Non‐COPD (n = 93)	COPD (n = 107)	*P*
Cough	10 (10.8)	16 (15.0)	0.378
Dyspnea	9 (9.7)	12 (11.2)	0.724
Hemoptysis	9 (9.7)	10 (9.3)	0.936
Fatigue	3 (3.2)	2 (1.9)	0.540
Fever	6 (6.5)	9 (8.4)	0.600
Neurologic symptom	1 (1.1)	5 (4.7)	0.137
Pain	4 (4.3)	8 (7.5)	0.346
Asymptomatic	51 (54.8)	45 (42.1)	0.071
Cardiovascular diseases	46 (49.5)	70 (65.4)	0.023
Hypertension	59 (63.4)	65 (60.7)	0.696
Diabetes	44 (47.3)	38 (35.5)	0.091
Cerebrovascular disease	20 (21.5)	38 (35.5)	0.029
Chronic renal disease	8 (8.6)	2 (1.9)	0.029
Hepatitis	3 (3.2)	7 (6.5)	0.283
Previous surgery within 5 y	25 (26.9)	36 (33.6)	0.3

Abbreviation: COPD, chronic obstructive pulmonary disease.

### 3.1 Survival analysis between the Non‐COPD and COPD Groups

**Figure 1:**
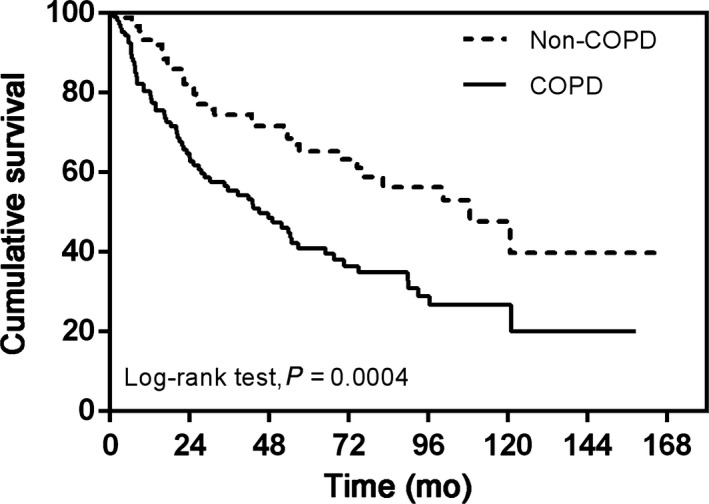
Kaplan–Meier curve of overall survival showing presence versus absence of chronic obstructive pulmonary disease 105 × 73 mm (300 × 300 DPI)

The cut‐off date for the final analysis was July 31, 2016. The median potential follow‐up times were 76.2 months and 71.7 months for the non‐COPD and COPD groups, respectively. All patients had a complete follow‐up. A total of 124/200 patients (45 [48.4%] in the non‐COPD group and 79 [73.8%] in the COPD group) died before the final analysis. The median overall survival was 108.5 months in the non‐COPD group and 45.0 months in the COPD group (HR: 2.05; 95% CI, 1.36‐2.97, *P* = 0.0004; Figure 1). The 1‐, 3‐, 5‐ and 10‐year estimated survival rates were 93.3%, 74.4%, 65.3%, and 39.7% in the non‐COPD group and 80.4%, 55.4%, 41.0%, and 20.0% in the COPD group, respectively.

**Figure 2:**
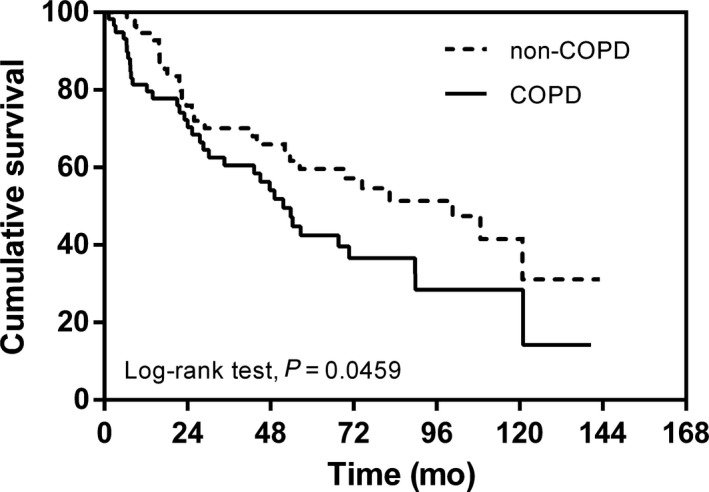
Kaplan–Meier curve of overall survival showing presence versus absence of chronic obstructive pulmonary disease after propensity score matching 105 × 73 mm (600 × 600 DPI)

A total of 118 patients underwent PSM between the two groups to yield 59 matched pairs. The general characteristics of the PSM patients were well‐balanced (Tables 3 and 4). The median survival times were 51.9 months in the PSM COPD group and 100.6 months in the PSM non‐COPD group; this difference was still significant (HR: 1.59; 95% CI, 1.096‐2.64, *P* = 0.0459; Figure 2).

**Table 3:** Demographic and clinical characteristics for PSM patients

Characteristics	Patients no (%)		
	Non‐COPD (n = 59)	COPD (n = 59)	*P*
Mean age ± SD	77.3 ± 8.5	78.3 ± 9.2	0.610
Range	56 ~ 91	54 ~ 101	
Age ≥75 y old			
Yes	39 (66.1)	40 (67.8)	0.845
No	20 (33.9)	19 (32.2)	
Gender			
Male	56 (94.9)	55 (93.2)	0.697
Female	3 (5.1)	4 (6.8)	
Smoking history			
Yes	37 (62.7)	33 (55.9)	0.453
No	22 (37.3)	26 (44.1)	
Smoking index, Pack‐years			
≥20	29 (49.2)	27 (45.8)	0.712
<20	30 (50.8)	32 (54.2)	
ECOG PS score			
0 ~ 1	29 (49.2)	25 (42.4)	0.460
≥2	30 (50.8)	34 (57.6)	
TNM stage			
I	37 (62.7)	36 (61.0)	0.877
II	4 (6.8)	3 (5.1)	
III	8 (13.6)	11 (18.6)	
IV	10 (16.9)	9 (15.3)	
Histologic subtype			
Squamous cell carcinoma	14 (23.7)	13 (22.0)	0.801
Adenocarcinoma	29 (49.2)	30 (50.8)	
Adenosquamous carcinoma	1 (1.7)	3 (5.1)	
NSCLC, NOS	13 (22.0)	10 (16.9)	
Others	2 (3.4)	3 (5.1)	
Initial treatment			
Surgery	22 (37.3)	19 (32.2)	0.760
Radiation	14 (23.7)	18 (30.5)	
Chemotherapy	6 (10.2)	5 (8.5)	
EGFR‐TKIs	10 (16.9)	7 (11.9)	
BSC	7 (11.9)	10 (16.9)	
Previous tumors			
Yes	42 (71.2)	45 (76.3)	0.530
No	17 (28.8)	14 (23.7)	

Abbreviations: BSC, best supportive care; COPD, chronic obstructive pulmonary disease; EGFR‐TKI, epidermal growth factor receptor‐tyrosine kinase inhibitor; PSM, propensity score matching.

**Table 4:** Symptoms at diagnosis and comorbidities for PSM patients

	Patients no (%)		
	Non‐COPD (n = 59)	COPD (n = 59)	*P*
Symptoms at diagnosis			
Cough	7 (11.9)	8 (13.6)	0.782
Dyspnea	7 (11.9)	4 (6.8)	0.342
Hemoptysis	7 (11.9)	2 (34)	0.083
Fatigue	2 (3.4)	2 (3.4)	0.691
Fever	6 (10.2)	5 (8.5)	0.752
Neurologic symptom	1 (1.7)	3 (5.1)	0.309
Pain	2 (3.4)	4 (6.8)	0.679
Asymptomatic	27 (45.8)	31 (52.5)	0.461
Comorbidities			
Cardiovascular diseases	36 (61.0)	33 (55.9)	0.575
Hypertension	40 (67.8)	41 (69.5)	0.843
Diabetes	25 (42.4)	22 (37.3)	0.573

COPD, chronic obstructive pulmonary disease; PSM, propensity score matching.

### 3.2 Univariate and multivariate analyses of risk factors for long‐term overall mortality

Univariate and multivariate analysis were preformed to clarify the risk factors for overall survival with a Cox proportional hazard model. The univariate analysis showed that presence of COPD, older age, positive smoking history, smoking index ≥20 pack‐years, ECOG PS ≥2, advanced disease stage (stages Ⅲ and Ⅳ), squamous cell carcinoma histological subtype, nonsurgery initial treatment, the presence of a cough, hemoptysis, neurological symptoms, cardiovascular disease, elevated serum CEA and NLR were predictors for shorter survival times. The multivariate analysis revealed that presence of COPD, older age, an advanced disease stage (stages Ⅲ and Ⅳ), squamous cell carcinoma histological subtype, the presence of a cough, elevated serum CEA and NLR remained significantly associated with shorter survival times (Table 5).

**Table 5:** Univariate and multivariate analysis of risk factors for overall survival with proportional hazard model

	Univariate analysis			Multivariate analysis		
	HR	95%CI	*P* value	HR	95%CI	*P* value
Smoking history						
No	Ref			Ref		
Yes	1.674	1.091 ~ 2.567	0.018	0.877	0.367 ~ 2.098	0.589
Smoking index						
<20	Ref			Ref		
≥20	1.685	1.133 ~ 2.505	0.010	1.008	0.461 ~ 2.202	0.942
ECOG PS score						
0 ~ 1	Ref			Ref		
≥2	1.709	1.47 ~ 2.548	0.008	1.107	0.471 ~ 2.604	0.717
Initial treatment						
Surgery	Ref			Ref		
Radiation	3.413	1.905 ~ 6.116	<0.0001	1.798	0.692 ~ 4.671	0.638
Chemotherapy	10.589	5.488 ~ 20.429	<0.0001	3.191	1.288 ~ 7.908	0.080
Target therapy	3.027	1.570 ~ 5.835	0.001	1.332	0.457 ~ 3.881	0.189
BSC	4.132	2.141 ~ 7.976	<0.0001	2.299	0.782 ~ 6.754	0.119
Hemoptysis	2.052	1.201 ~ 3.507	0.009	1.165	0.569 ~ 2.385	0.092
Neurologic symptom	3.501	1.325 ~ 7.927	0.009	2.497	0.826 ~ 7.551	0.113
Cardiovascular disease	1.763	1.158 ~ 2.684	0.008	1.089	0.628 ~ 1.888	0.397
Presence of COPD						
Non‐COPD	Ref			Ref		
COPD	2.059	1.362 ~ 3.115	0.001	1.619	1.098 ~ 2.314	0.030
Age	1.049	1.024 ~ 1.075	<0.001	1.077	1.050 ~ 1.105	<0.0001
TNM stage						
Ⅰ	Ref			Ref		
Ⅱ	0.493	0.118 ~ 2.059	0.332	0.609	0.134 ~ 2.755	0.519
Ⅲ	4.583	2.862 ~ 7.339	<0.0001	5.513	3.306 ~ 9.196	<0.0001
Ⅳ	6.886	4.111 ~ 11.536	<0.0001	11.743	6.507 ~ 21.191	<0.0001
Histology subtype						
Non‐SCC	Ref			Ref		
SCC	2.358	1.517 ~ 3.663	<0.0001	3.106	1.949 ~ 4.926	<0.0001
Cough	1.984	1.171 ~ 3.362	0.011	2.463	1.415 ~ 4.286	0.001
Serum level of CEA, ng/ml	1.001	1.000 ~ 1.002	0.005	1.001	1.000 ~ 1.001	0.023
Neutrophil‐Lymphocyte Ratio	2.452	1.837 ~ 3.114	<0.0001	2.615	1.476 ~ 4.832	0.007

Abbreviations: BSC, best supportive care; CEA, carcinoembryonic antigen; COPD, chronic obstructive pulmonary disease; ECOG PS, Eastern Cooperative Oncology Group performance status; NLR, neutrophil‐lymphocyte ratio.

## 4 DISCUSSION

In clinical practice, NSCLC patients who also have COPD are very common, especially in the geriatric ward. The mechanistic links between lung cancer and COPD have been widely investigated. Many studies^14^, ^15^, ^16^, ^17^, ^18^, ^19^ and reviews^20^, ^21^ have shown how key pathological features of airway and airspace disease in COPD develop to lung cancer gradually. The purpose of this study was to explore the clinical impact of COPD on the long‐term survival of patients with NSCLC as well as other clinical features related to the presence of COPD. Our data indicated that the patients with non‐COPD NSCLC had significantly better overall survival than the patients with COPD‐NSCLC in the unmatched or PSM comparisons. The multivariate analysis for all patients (n = 200) also showed that co‐existing COPD lead to a significant harmful effects for the mortality of NSCLC patients, and COPD was a prognostic factor for poorer survival in this group of patients.

Several other studies had investigated and demonstrated the impact of COPD in the prognosis of NSCLC.^12^, ^13^, ^22^, ^23^ Although they had found that COPD was related to a poorer prognosis in NSCLC, which were similar to the results of our study, an increasing number of other studies (eg, the studies performed by Lee,^9^ Gullon^10^ and Mina^11^) had reported conflicting results for the impact of COPD on survival. In another study, comorbidities including COPD did not predict a worse outcome for 57 elderly advanced lung cancer patients who were poor candidates for platinum‐based therapy and were treated weekly with paclitaxel. Ueda et al^24^ reported that computed tomography diagnosed emphysema but not the degree of airway obstruction was associated with a poor prognosis. These results were consistent with Gullon's^10^ study. Although previous similar studies conflicted and made drawing conclusions about whether COPD was a prognostic factor for NSCLC difficult, our findings supported the view that presence of COPD was a poor prognosis factor for survival in this group of NSCLC. Moreover, the mOS in non‐COPD group was almost 2 times longer than that of the COPD group (100.6 m vs 51.9 m).

COPD patients had a worse survival may be related to the impact of comorbidities. It is the common sense that cigarette smoking is closely related to many diseases, some of which are lethal. There were more heavy smokers in CODP group, and they were more prone to cardiovascular, cerebrovascular diseases and chronic renal disease. A total of 17 patients in COPD group died of noncancer causes, including pneumonia, heart disease, renal failure and diabetes related soft tissue infection. They were all heavy smokers with more than 60 pack‐year cigarettes smoking. In the univariate analysis, we had concluded that presence of neurologic symptom and cardiovascular disease had a margin statistical significance related to the increasing risk of death.

Our study showed that squamous cell lung cancer was another independent negative prognostic factor for overall survival. One possible explanation may be attributed to the wide use of EGFR‐TKI (epidermal growth factor receptor‐tyrosine kinase inhibitor) or ALK‐TKI (anaplastic lymphoma kinase) in clinical practice. EGFR mutations and ALK rearrangements have been recognized as driver genes, and several small molecule agents have been developed to attack these targets, leading to significant clinical improvements in these patient populations.^25^, ^26^, ^27^, ^28^ Demographic and subgroup analyses showed that mutations in these genes were more common in lung adenocarcinoma and less common in squamous cell carcinoma.^29^, ^30^, ^31^ The therapeutic options and alternative agents for squamous cell carcinoma are reduced compared to adenocarcinoma. Many attempts have been made to develop new therapies for squamous cell carcinoma, although progress has been slow.^32^, ^33^, ^34^ In recent years, several checkpoint inhibitors have showed powerful anti‐cancer effects. Nivolumab is the first PD‐1 inhibitor approved for the treatment of advanced‐stage squamous cell NSCLC following platinum‐based chemotherapy. In the key Phase III trial CHECKMATE 017, a better overall survival (9.2 m vs 6.0 m HR 0.59, 95%CI 0.44‐0.79, *P* < 0.001) were seen in patients treated with second‐line nivolumab compared with docetaxel.^35^ Unfortunately, this group of patients did not use these drugs. Another possible reason for the significantly higher number of patients with stage Ⅲ and Ⅳ squamous cell carcinoma (39.1%) compared to those with nonsquamous cell carcinoma (25.8%) is that central type lung cancer is more common in patients with squamous cell carcinoma.

Cough is one of the major symptoms of lung cancer and seriously affects the quality of life (QoL). The description from the American College of Clinical Pharmacy (ACCP) evidence‐based clinical practice guidelines indicates that a cough is more likely to indicate airway involvement than lung parenchyma involvement due to the location of the cough receptors.^36^ One possible reason for the cough as a negative prognostic factor may be that management of a cough in lung cancer has a lower priority than the management of other cancer symptoms.^37^ The decline in the QoL caused by cough has a great impact on survival. Sakurai et al^38^ prospectively assessed the cough‐related QoL in patients with lung cancer using the Leicester Cough Questionnaire (LCQ). The multivariate logistic regression analysis showed that the presence of endobronchial lesions was an independent predictor of an impaired cough‐related QoL. Molassiotis et al^39^ highlighted the complex and distressing nature of cough, including its interaction with other symptoms, such as dyspnea, fatigue, and sleep disorders. Coughing in daily life impacts socialization by causing embarrassment and other psychological effects. A second possible reason is that a cough is a nonspecific symptom that is present in many respiratory diseases other than NSCLC. Therefore, clinicians do not give a cough the same attention as other cancer symptoms. The diagnosis of NSCLC may be delayed when a mild cough is the only presenting symptom. Although more attention has been given to improving the assessment and management of cough, a fully validated tool should be tested in the clinical setting.^40^

The serum CEA level is the most commonly used clinical tumor markers. A number of studies reported that elevated CEA was related to advanced disease (in terms of the p‐stage) and poor survival.^41^ CEA is an independent predictor for DFS and OS in EGFR wild‐type adenocarcinoma and the L858R substitution subgroup^42^ and a major prognostic predictor for EGFR mutation‐positive NSCLC with intracranial metastases when using brain radiotherapy and EGFR‐TKI.^43^ For the entire cohort, elevated CEA serum levels were a significant prognostic factor for a shorter survival time. We separated the patients into two groups using a cut‐off value of 20 ng/mL or a more stringent cut‐off value of 10 ng/mL and found that higher CEA levels were still significantly associated with poorer OS in the proportional hazards model (data not shown).

More recently, an elevation in the NLR, an indicator of inflammation, has been associated with poor prognosis in various diseases.^44^, ^45^, ^46^ It has been recently reported that the NLR could be considered to be a marker for COPD patients in assessing inflammation because the NLR was higher in exacerbated than in stable patients.^47^ Besides that, in lung cancer, the elevated pretreatment NLR is also be proved as a poor prognostic factor.^48^ In a retrospective cohort of 175 patients treated with nivolumab, NLR of 5 or greater was associated with poor prognosis.^49^ In our study, we found that serum NLR level were significantly higher in COPD‐NSCLC than in patients without COPD and this significance remained even after PSM. This result suggested that when COPD co‐existed with NSCLC, which were both closely related to chronic inflammation, the increase in neutrophils and reduction in lymphocyte would be more prominent. Lymphocyte is one of the main members of tumor immune, which is involved in the recognition, attack, and apoptosis of tumor cells. The decrease of the proportion of lymphocytes means that the body's immune system was weakened and suppressed, which predicts poor prognosis. As a result of this study, elevated NLR is an independent risk factor for poor prognosis in this group of patients.

Our study reports the 15‐year survival outcomes of patients with NSCLC with and without COPD. Although the follow‐up time was long, several limitations that are inherent to a retrospective analysis must be considered. While we used a PSM program, reducing bias between groups similar to prospective randomized studies is impossible. Because more than half of the patients in the current study are over 80 years of age, these elderly people may not properly perform pulmonary function tests, which may lead to potential false positive results when diagnosing COPD. Additionally, relatively few patients were included in the study due to the high number of ineligible patients (n = 101) and because this was a single center study. We found 80 patients who did not have pulmonary function measurements at baseline, and their general conditions were considered inadequate, suggesting that the management of NSCLC needs improvement in the future.

In conclusion, elderly patients with NSCLC and COPD had worse survival outcomes than patients without COPD. This indicates that pulmonary function tests in elderly lung cancer patients are particularly important, and we should pay more attention on these COPD‐NSCLC coexistence patients. Additional prospective studies with more patients are necessary to fully clarify the relationship between COPD and NSCLC.
